# Spatiotemporal Heterogeneity Analysis of Hemorrhagic Fever with Renal Syndrome in China Using Geographically Weighted Regression Models

**DOI:** 10.3390/ijerph111212129

**Published:** 2014-11-25

**Authors:** Shujuan Li, Hongyan Ren, Wensheng Hu, Liang Lu, Xinliang Xu, Dafang Zhuang, Qiyong Liu

**Affiliations:** 1 State Key Laboratory of Resources and Environmental Information System, Institute of Geographical Sciences and Natural Resources Research, Chinese Academy of Sciences, 11A Datun Road, Chaoyang District, Beijing 100101, China; E-Mails: lishujuan711@126.com (S.L.); xuxl@lreis.ac.cn (X.X.); zhuangdf@lreis.ac.cn (D.Z.); 2 College of Resources and Environment, University of Chinese Academy of Sciences, No. 19 Yuquan Road, Beijing 100049, China; 3 Center for Health Statistics and Information, National Health and Family Planning Commission, No.38 Beilishi Road, Xicheng District, Beijing 100044, China; E-Mail: huws@moh.gov.cn; 4 State Key Laboratory for Infectious Diseases Prevention and Control, National Institute for Communicable Disease Control and Prevention, China CDC, 5 Changbai Road, Changping, Beijing 102206, China; E-Mail: liuqiyong@icdc.cn

**Keywords:** hemorrhagic fever with renal syndrome (HFRS), geographically weighted regression (GWR), spatiotemporal heterogeneity, affecting factors

## Abstract

Hemorrhagic fever with renal syndrome (HFRS) is an important public health problem in China. The identification of the spatiotemporal pattern of HFRS will provide a foundation for the effective control of the disease. Based on the incidence of HFRS, as well as environmental factors, and social-economic factors of China from 2005–2012, this paper identified the spatiotemporal characteristics of HFRS distribution and the factors that impact this distribution. The results indicate that the spatial distribution of HFRS had a significant, positive spatial correlation. The spatiotemporal heterogeneity was affected by the temperature, precipitation, humidity, NDVI of January, NDVI of August for the previous year, land use, and elevation in 2005–2009. However, these factors did not explain the spatiotemporal heterogeneity of HFRS incidences in 2010–2012. Spatiotemporal heterogeneity of provincial HFRS incidences and its relation to environmental factors would provide valuable information for hygiene authorities to design and implement effective measures for the prevention and control of HFRS in China.

## 1. Introduction

Hemorrhagic fever with renal syndrome (HFRS), a rodent-born endemic disease caused by hantaviruses (family Bunyaviridae), has a wide global distribution. China is the most severely hantavirus-affected country and has accounted for 90% of global HFRS cases in the last decade. Moreover, there is a tendency of HFRS prevalence in the autonomous regions and metropolitan areas, with the exception of Qinghai and Taiwan Provinces [1]. During previous decades, the overall HFRS incidence has declined considerably in mainland China [2]. However, in recent years, the HFRS incidence has tended to increase in some regions of China [2,3]. It is necessary to identify the specific regions and potential factors that comprise this distribution.

In China, HFRS is caused mainly by two types of hantavirus, Hantaan virus (HTNV) and Seoul virus (SEOV), each of which has co-evolved with a distinct rodent host. HTNV is carried by *Apodemusagrarius*, and SEOV by *Rattusnorvegicus*. Rodents are the predominant reservoir of hantavirus and excrete virus-containing urine, feces, and saliva when chronically infected. Humans usually become infected with hantaviruses through contact with or inhalation of aerosols and secretions from infected rodent hosts [4].The external environmental factors, including temperature, rainfall, relative humidity, NDVI, elevation and land use, not only affect the rate of replication of virus, but also have an impact on disease reservoir-rodents and contact between the human and rodent populations [5], which eventually affects HFRS incidence.

Studies in different areas of China and other countries have suggested that external environmental factors, including natural factors (such as temperature [6,7,8], precipitation [4,5,9], humidity [7], normalized difference vegetation index (NDVI) [10,11] and elevation [6]) and social-economic factors (such as land use [6,12]), may affect the incidence of HFRS. These factors may differentially influence the incidence of HFRS in different regions because of the spatiotemporal heterogeneity in climate types, ecological characteristics, population immunity, public health intervention measures, and socioeconomic factors within different regions [13].

Many studies have been conducted regarding the pathogenesis of hantaviruses, the epidemiologic characteristics of HFRS, and the potential affecting factors [3,6,10,14,15,16]; these studies have facilitated an understanding of the severity of HFRS and its spatial distribution in China [12,17,18,19,20,21]. However, only a few studies have investigated the impact of natural and social-economic factors on the spatiotemporal heterogeneity of HFRS. The distribution of HFRS has changed in recent years, and the reasons for this change remain unclear. It is important to characterize the HFRS spatial-temporal trend and reveal the potential affecting factors. Given the spatiotemporal heterogeneity of HFRS cases, the geographically weighted regression (GWR) model serves a good means of finding the local factors affecting HFRS epidemics. The objective of this study was to characterize the spatiotemporal dynamics of HFRS epidemics and to identify the impact of environmental factors and social-economic factors for the spatiotemporal heterogeneity with the GWR model. This study should provide valuable information for health authorities to design and implement effective measures for the prevention and control of HFRS in China.

## 2. Material and Methods

### 2.1. Data Collection

Data regarding the HFRS incidence, population, environmental factors, and social-economic factors at the province level of China in 2005–2012 were collected. The environmental factors [22] included temperature [6,7], precipitation [6,23], humidity [7], NDVI [10], and elevation [9,24]. The social-economic factors included land use [6], cultivated land area and grain yield. The specific variables used are listed in Table 1.

**Table 1:** List of variables used in the HFRS incidence analysis in China, 2005 to 2012.

Variables	Type and Year	Data Source
Temperature	Yearly mean temperature	China Meteorological Data Sharing Service System
Precipitation	Yearly mean temperature	China Meteorological Data Sharing Service System
Humidity	Yearly mean temperature	China Meteorological Data Sharing Service System
NDVI	Yearly mean temperature	ftp://ladsweb.nascom.nasa.gov/
NDVI01	Monthly mean NDVI of January	ftp://ladsweb.nascom.nasa.gov/
NDVI08	Monthly mean NDVI of January	ftp://ladsweb.nascom.nasa.gov/
Cultivatedland area	Acreage sown to grain	China Statistical Yearbook
Grain yield	Grain production	China Statistical Yearbook
Land50	Closed (>40%) broad-leaved deciduous forest (>5 m)	http://due.esrin.esa.int/globcover/
Land100	Closed to open (>15%) mixed broad-leaved and needle-leaved forest (>5 m)	http://due.esrin.esa.int/globcover/
Land110	Mosaic forest or shrub-land (50–70%)/grassland (20–50%)	http://due.esrin.esa.int/globcover/
Land120	Mosaic grassland (50–70%)/forest or shrub-land (20–50%)	http://due.esrin.esa.int/globcover/
Elevation	DEM data	Data Center for Recourses and Environmental Sciences Chinese Academy of Sciences

The epidemiologic data from 31 provinces in mainland China were provided by the China Information System for Disease Control and Prevention. Because of the limitations of the data sources, the CDC records were insufficient to distinguish whether the reported numbers of human HFRS cases were caused by HNTV or by SEOV. However, according to Zhang’s study, from 2006 to 2012, HFRS infection in China was mainly caused by *Apodemus,* and the proportion of *Apodemus*-type infection in China had been increasing [25], so in the study, we did not distinguish HFRS cases caused by HNTV or by SEOV. Meteorological data as yearly means of precipitation, temperature and humidity were obtained from the China Meteorological Data Sharing Service System. Population and social-economicfactors (including cultivatedland area and grain yield) were obtained from the China Statistical Yearbook. Geographical data, including administrative data and Digital Elevation Model (DEM), were provided by the Data Center for Recourses and Environmental Sciences Chinese Academy of Sciences. NDVI data (including yearly mean NDVI and monthly mean NDVI) were obtained from ftp://ladsweb.nascom.nasa.gov/. Land use data (resolution of 300 m) were obtained from http://due.esrin.esa.int/globcover/.

### 2.2. Methods

#### 2.2.1. Spatial Auto-Correlation

Spatial auto-correlation [26] measures the degree of dependency among events while simultaneously considering their similarities and distance relationships [27,28,29].

(1) Global Indicators of Spatial Auto-Correlation

Global indicators of auto-correlation measure if and how much a dataset is auto-correlated throughout the study region. One of the principal global indicators of auto-correlation is Moran’s index I [28], which is defined in Equation (1):

(1) $$Moran^{'}s\;I=\frac{N}{S}\cdot \frac{\sum_{i=1}^{N}\sum_{j=1}^{N}w_{ij}\left(Y_{i}-\bar{Y}\right)\left(Y_{j}-\bar{Y}\right)}{\sum_{i=1}^{N}{\left(Y_{i}-\bar{Y}\right)}^{2}}$$

where *N* is the total pixel number, and in this study *N* refers to 31, the number of study provinces; *Y_i_* and *Y_j_* are the attribute value at points *i* and *j* (with *i* ≠ *j*), and in the study *Y_i_* refers to the HFRS incidence at province *i*; $\bar{Y}$ is the average value of HFRS incidence; *w_ij_* is an element of the weight matrix (N × N). *w_ij_* is a weight which can be defined as follows: when location *i* is contiguous to location *j*, the weight *w_ij_* is given the weight of 1, otherwise the *w_ij_* is given the weight of 0. $S=\overset{N}{\underset{i=1}{\sum }}\overset{N}{\underset{j=1}{\sum }}w_{\text{ij}}.$ I∈[−1,1]. I∈[−1,0), there is a negative auto-correlation; if I∈(0,1], there is a positive auto-correlation; if *I*=0, there is no auto-correlation.

(2) Local Indicators of Spatial Auto-Correlation (LISA)

Local indicators of spatial auto-correlation (LISA) enable the localization of clustered pixels by measuring the number of features inside the fixed neighborhood that are homogeneous [27,30]. In this study, we used the Local Moran’s I formula as defined below in Equation (2):

(2) $$I_{i}=Z_{i}\overset{N}{\underset{j=1}{\sum }}w_{ij}Z_{j}$$

where $Z_{i}=\frac{\left(Y_{i}-\bar{Y}\right)}{\sqrt{\frac{1}{N}\sum_{i=1}^{N}{\left(Y_{i}-\bar{Y}\right)}^{2}}}$ and $Z_{j}=\frac{\left(Y_{j}-\bar{Y}\right)}{\sqrt{\frac{1}{N}\sum_{j=1}^{N}{\left(Y_{j}-\bar{Y}\right)}^{2}}}$ are the standardized intensities at points *i* and *j* (*i* ≠ *j*) and *w_ij_* is an element of the weight matrix.

A high positive local Moran’s I value implies that the location has similarly high or low values as its neighbors, thus the locations are spatial clusters. Spatial clusters include “High-High” clusters (high values in a high value neighborhood) and “Low-Low” clusters (low values in a low value neighborhood).A high negative local Moran’s I value means that the location under study is a spatial outlier. Spatial outliers are those values that are obviously different from the values of their surrounding locations. Spatial outliers include “High-Low” (a high value in a low value neighborhood) and “Low-High” (a low value in a high value neighborhood) outliers [31].

#### 2.2.2. Geographically Weighted Regression (GWR) Model

Given the spatiotemporal heterogeneity of HFRS, the related factors may affect HFRS in different ways and to different degrees, which is appropriate to analyze using a GWR model. Geographically weighted regression is an extension of the traditional multiple linear regression toward a local regression in which the regression coefficients are specific to a location rather than global estimates [26,27]. The geographically weighted regression (GWR) model is based on the spatial non-stationarity, which is common in spatial process: an explanation might be highly relevant in one application, but seemingly irrelevant in another; parameters describing the same relationship might be negative in some applications but positive in others; and the same model might replicate data accurately in one system but not in another [32]. A GWR model embeds the data’s spatial location into the regression parameter [32]. The local estimation of the parameters with GWR is expressed by Equation(3) [33]:

(3) $$y_{i}=\beta_{0}\left(u_{i},v_{i}\right)+\overset{n}{\underset{k=1}{\sum }}\beta_{ik}\left(u_{i},v_{i}\right)x_{ik}+\varepsilon_{i}$$

*I* = 1, 2, …, *m*

where *i* = 1, 2, …, 31 denotes the spatial location of provinces in China; *y_i_* is the dependent variable HFRS incidence at location *i*; independent variables *x_ik_* is the value of the *k* parameter at location *i*, and in this study *x_ik_* referred to the value of an affecting factor *k* (such as temperature, precipitation, NDVI) at province *i*, which is specific for every province; β_*0*_ is the intercept; β_*ik*_ is the correlation coefficient for the independent predictor variable *x_ik_*, which is to be estimated; and $\varepsilon_{i}$ represents random error. Therefore every province in our study has a set of specific parameters to reflect the relationship between HFRS incidence and affecting factors. The regression coefficients of this equation are estimated at each location using data within a neighborhood. Therefore, this GWR model can measure the spatial variations in relationships [34].

### 2.3. Data Analyses Using Computer Software

The calculation of spatial clusters and spatial outliers was performed using the software GeoDa (version 1.6.6, Spatial Analysis Laboratory, Urbana, IL, USA, 2014). Spatial analysis and GWR model analysis were performed using the software ArcGIS10.1 (ESRI, Redlands, CA, USA).

## 3. Results and Discussion

### 3.1. Descriptive Statistics

**Figure 1:**
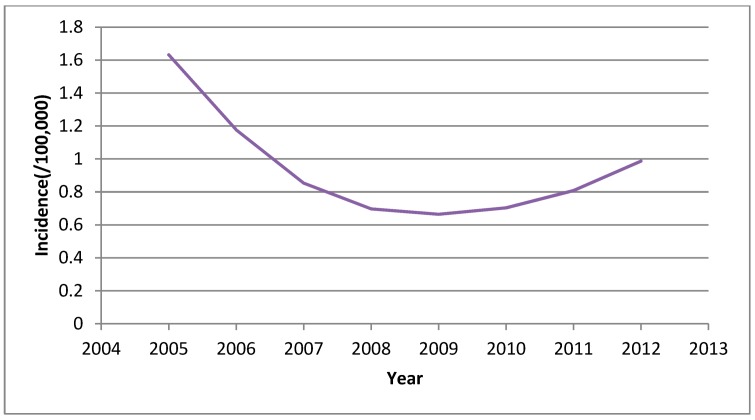
The incidence of HFRS in China, 2005–2012.

In 2005–2012, the epidemic situation presented an initial decline, which was followed by a slight increase (Figure 1). The incidence was 1.63/100,000 in 2005, declined to 0.66/100,000 in 2009, and then increased to 0.99/100,000 in 2012.The declining trend prior to 2009 fits well with the investment in public health and the improvement in health care and quality of life during these years [14]. Some efforts should be made to define the factors contributing to the increasing trend of HFRS incidence since 2009.

**Figure 2:**
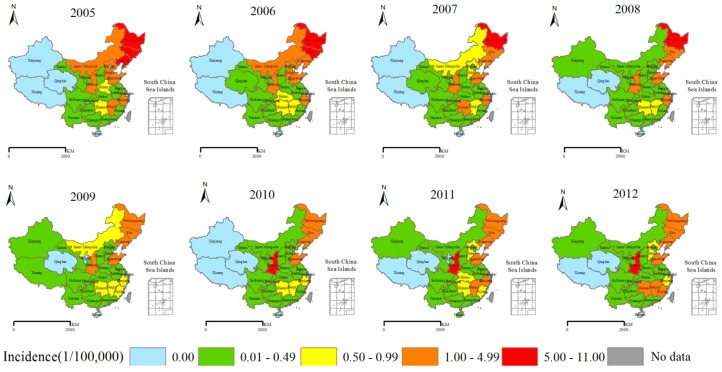
Yearly distribution of HFRS incidence in China, 2005–2012. * Per 100,000 individuals.

The HFRS incidence varied between provinces (Figure 2). In 2005–2009, Heilongjiang, Jilin, Liaoning, Shandong, Inner Mongolia, Shaanxi, and Zhejiang presented higher incidences; except for Shaanxi, these provinces with high HFRS incidences showed a declining trend. From 2010–2012, Shaanxi surpassed Heilongjiang and became the province with the highest HFRS incidence; however, the incidence in Shaanxi Province was not higher than the incidences in Heilongjiang and Liaoning in 2005 and 2006. The HFRS incidences of traditional HFRS epidemic area in northeast China declined, which maybe the result of large-scale vaccination campaigns [25,35].

### 3.2. Correlation Analysis

In this study, we adopted Pearson’s correlation coefficient to measure the correlation between HFRS incidence and possibly affecting factors. Pearson’s correlation coefficient is a measure that determines the degree to which two variable’s movements are associated. Many factors affected the HFRS incidence. According to Yan’s study, the peak HFRS frequency occurred three or four months later than the monthly NDVI peak [10]. Considering the results from Yan’s study [10] and the seasonal characteristics of HFRS, the correlation between the annual HFRS incidence and the yearly mean NDVI and monthly mean NDVI were considered. According to the results (Table 2), temperature, NDVI of January, grain yield, and land use (Land50/Land100/Land110/Land120) were strongly correlated; the other factors were not significantly correlated.

This result might be because of the large scale of HFRS incidence. The correlation analysis also indicated that the relative factors of HFRS incidence varied with years.

**Table 2:** Correlations between HFRS incidence and potential related factors.

Potential Related Factors	2005	2006	2007	2008	2009	2010	2011	2012
Temperature	−0.390 *	−0.396 *	−0.298	−0.257	−0.314	−0.229	−0.19	−0.169
Precipitation	−0.226	−0.189	−0.206	−0.208	−0.137	−0.084	−0.077	−0.092
Humidity	−0.077	−0.061	−0.006	−0.018	0.063	0.106	0.121	0.064
NDVI	−0.075	−0.047	0.003	0.026	0.004	0.023	0.048	0.056
NDVI01	−0.389 *	−0.313	−0.185	−0.207	−0.205	−0.175	−0.092	−0.056
NDVI08	0.279	0.301	0.297	0.269	0.294	0.245	0.258	0.182
Cultivated land area	0.237	0.307	0.342	0.288	0.325	0.228	0.18	0.157
Grain yield	0.355 *	0.399 *	0.380 *	0.356 *	0.335	0.262	0.271	0.235
Land50	0.612 **	0.738 **	0.811 **	0.727 **	0.695 **	0.486 **	0.460 **	0.381 *
Land100	0.645 **	0.758 **	0.776 **	0.173	0.18	0.139	0.166	0.131
Land110	0.756 **	0.847 **	0.861 **	0.720 **	0.702 **	0.481 **	0.444 *	0.365 *
Land120	0.496 **	0.584 **	0.613 **	0.452 *	0.448 *	0.338	0.308	0.266
Elevation	−0.243	−0.234	−0.229	−0.212	−0.216	−0.161	−0.164	−0.141

Notes: * represents *p* < 0.05; ** represents *p* < 0.01.

### 3.3. Spatiotemporal Heterogeneity

Based on the statistical and spatial analyses of HFRS incidence, the spatial auto-correlation was analyzed (Table 3). The HFRS incidence was significantly and positively auto-correlated in 2005–2009. The Global Moran’s *I* scores were above 0.21 (*p* < 0.03), and the Global Moran’s *I* scores from 2010–2012 significantly declined. The HFRS incidence was clustered from 2005–2009 and was then randomly distributed from 2010–2012.

**Table 3:** Global Moran’s *I*.

Year	Moran’s *I*	*p*
2005	0.50	<0.01
2006	0.45	<0.01
2007	0.28	<0.01
2008	0.21	0.03
2009	0.26	0.02
2010	0.05	0.18
2011	0.00	0.29
2012	−0.04	0.46

**Figure 3:**
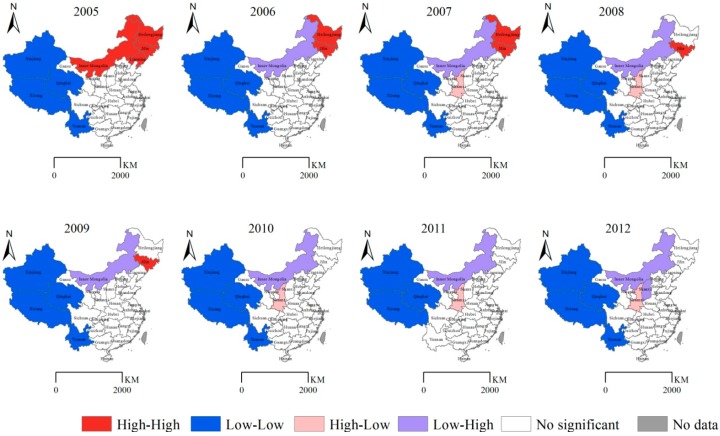
Yearly LISA cluster maps of hemorrhagic fever with renal syndrome (HFRS) in the People’s Republic of China, 2005–2012. * Per 100,000 individuals.

Local indicators of spatial auto-correlation (LISA) can reflect the spatial clustering. The yearly LISA cluster maps of HFRS (Figure 3) demonstrated that Heilongjiang, Jilin, Liaoning, and Inner Mongolia constituted “High-High” zones in 2005.

In 2006 (in contrast to 2005), Inner Mongolia became a “Low-High” zone. In 2007 (in contrast to 2006), Shaanxi transitioned to a “High-Low” zone. In 2008 (in contrast to 2007), Jilin separately composed a “High-High” zone. In 2009 (in contrast to 2008), Shaanxi was not different from the surrounding provinces. From 2010–2012, Shaanxi constituted an obvious “High-Low” zone. The results demonstrate that since 2010, the traditional HFRS epidemic area in Northeast China appeared to be random, and Shaanxi became an HFRS “hot spot”, which indicated that HFRS incidence in Shaanxi exceeded the neighboring provinces’.

### 3.4. Correlation between HFRS Spatiotemporal Heterogeneity and Related Factors

#### 3.4.1. 2005–2009: GWR Modeling and Spatiotemporal Heterogeneity Cause Analysis

The correlation analysis indicated the HFRS incidences were significantly correlated with temperature, NDVI of January, grain yield, and land use (Land50/Land100/Land110/Land120), but not significantly with others, which meant the HFRS incidences were globally associated with the significant variables, but not globally associated with the others. The other factors were affecting factors chosen in many studies [5,9,20,36,37] and were significantly associated with HFRS incidence at some local scales. Exclusion of these factors might lead to losing potential information affecting HFRS epidemics.

Based on the correlation analysis and other studies [10,13,20,22], the factors chosen for the GWR model included temperature, precipitation, humidity, NDVI01 (NDVI of January), NDVI08 (NDVI of August for the previous year), cultivated land area, grain yield, Land50, Land100, Land110, Land120 and elevation.

The GWR models of the HFRS incidence and related factors were constructed on the basis of the spatiotemporal heterogeneity of HFRS incidence rates. In this study, we have chosen an ADAPTIVE kernel whose bandwidth will be found by minimizing the corrected Akaike Information Criterion (AICc) value, which attempts to identify the best fixed distance or the best appropriate number of adjacent points of the regression province. The optimal GWR model for different year was chosen by the highest R^2^ value, which indicated the HFRS incidence was explained to a maximum extent. The results of the GWR model were compared with the results of an OLS model to identify the best interpretation for the spatiotemporal heterogeneity of the HFRS incidence rates. The R^2^ values obtained from the GWR model were between 0.60–0.88 in 2005–2009, and the R^2^ values obtained from the OLS model were between 0.41–0.84.The AICc values with the GWR model were lower than the AICc values with the OLS model (Table 4). The R^2^ values with the GWR model first increased and then decreased from 2005–2009 (Table 4).

The results from the GWR model indicated a significant improvement compared with the OLS model, which could indicate that the GWR model explained more of the spatiotemporal heterogeneity. The GWR models for different years differed in their abilities to explain the spatiotemporal heterogeneity of HFRS.

**Figure 4:**
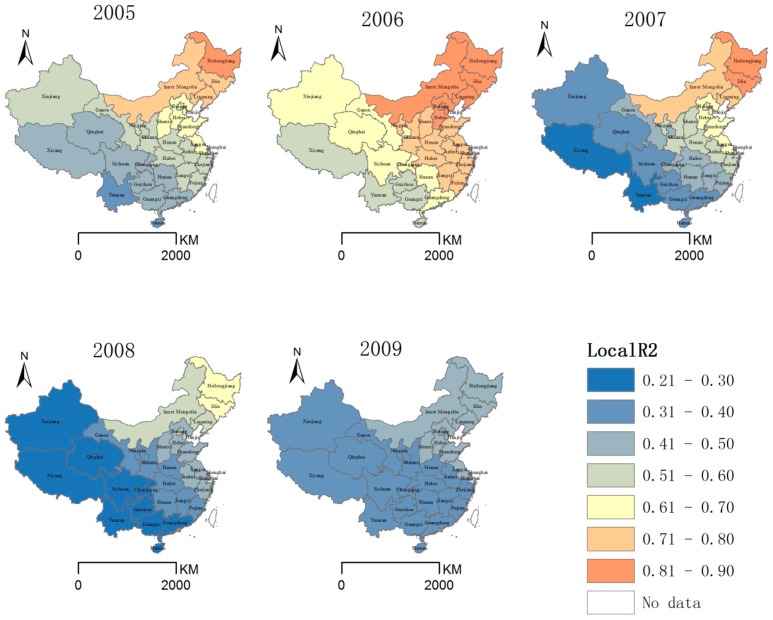
Distribution of GWR local R^2^ values, 2005–2009.

The R^2^ values exhibited a dissimilar distribution within each province in different years (Figure 4). In 2005–2007, the R^2^ values were relatively higher than the values in 2008 and 2009. The values in 2005–2009 were 38–82%, 51–90%, 26–84%, 21–68%, and 33–47%, respectively. In 2005–2007, the R^2^ values varied among the provinces, and the spatial distribution of the R^2^ values revealed a general decreasing trend from northeast to southwest, which indicated the factors of the GWR model explained the HFRS incidence well and the ability to explain the HFRS incidence was better in the traditional HFRS epidemic area in Northeast China compared with other areas.

**Table 4:** Comparison of the OLS and GWR models.

Year	Model	AICc	R^2^	R^2^ adjusted	*p*
2005	OLS	−583.57	0.70	0.66	0.000
2005	GWR	−591.09	0.81	0.76	<0.001
2006	OLS	−617.29	0.84	0.81	0
2006	GWR	−619.38	0.88	0.84	<0.001
2007	OLS	−620.36	0.69	0.66	0.000
2007	GWR	−628.60	0.82	0.76	<0.001
2008	OLS	−621.64	0.48	0.42	0.001
2008	GWR	−616.35	0.60	0.44	<0.001
2009	OLS	−622.18	0.41	0.34	0.004
2009	GWR	−627.27	0.63	0.55	<0.001

The differences in the explanatory ability of the GWR model might be caused by differences in the HFRS incidences within different areas. This result is supported by the fact that the GWR model performed better in Northeast China (higher incidence) than Southwest China (lower incidence). In 2008 and 2009, the spatial differences in the R^2^ values were not obvious, which indicated there might be other factors not considered in this study.

Different factors were chosen for the different optimal GWR models in different years(Table 5), and the coefficients (slope of variables) for specific year were different, which indicated that some factors affected the HFRS incidence in some years while some others did not, and that some factors weighed more than others in explaining the incidence. In 2005–2009, temperature, the NDVI of August for the previous year, and elevation were chosen for the model three times; precipitation and Land120 were chosen for the model twice; humidity, NDVI of January, and Land110 were chosen once. Additionally, different factors performed differently in the models. Temperature, precipitation and elevation decreased the HFRS incidence, while other factors promoted the HFRS incidence.

**Table 5:** The GWR model coefficients, 2005–2009.

Year	Temperature 10E−6	Precipitation 10E−7	Humidity 10E−7	NDVI01 10E−9	NDVI08 10E−9	Elevation 10E−9	Land110 10E−11	Land120 10E−10
2005	−6.93–−2.29				3.20−12.31	−19.92–−8.69		
2006	−2.95–−1.21				1.82−8.41	−6.52–−5.67	3.75−6.57	
2007	−7.03–−1.97			3.28−14.08		−26.11–−7.23		
2008		−1.25–−0.64			2.95−5.25			0.37−1.95
2009		−2.00–−1.55	8.41−10.39					1.14−1.50

**Figure 5:**
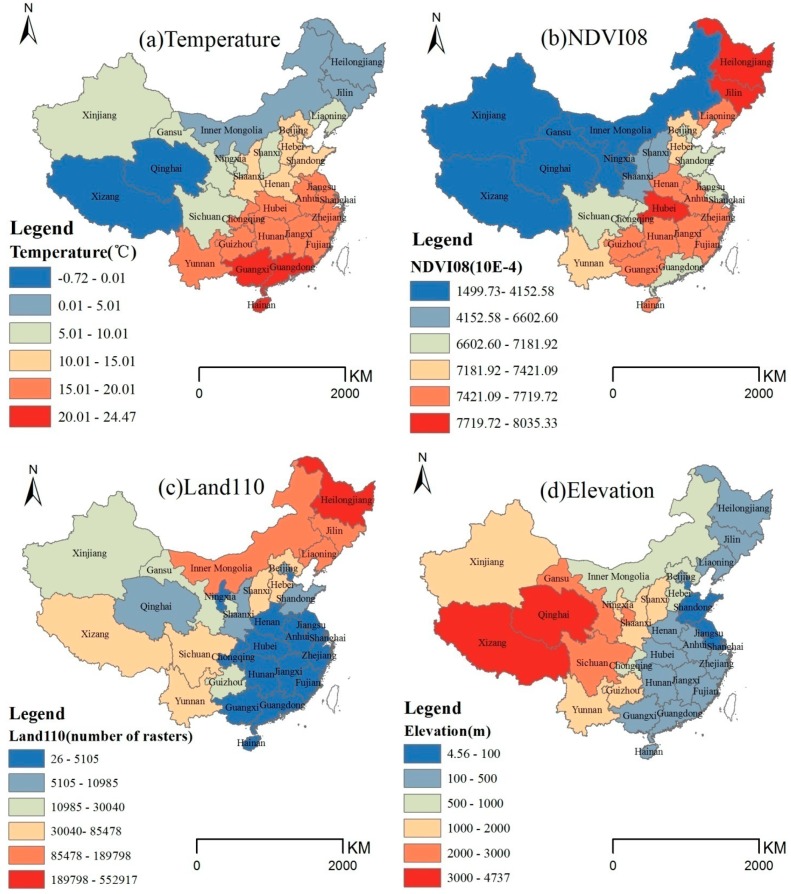
Independent factor distribution, 2006.

**Figure 6:**
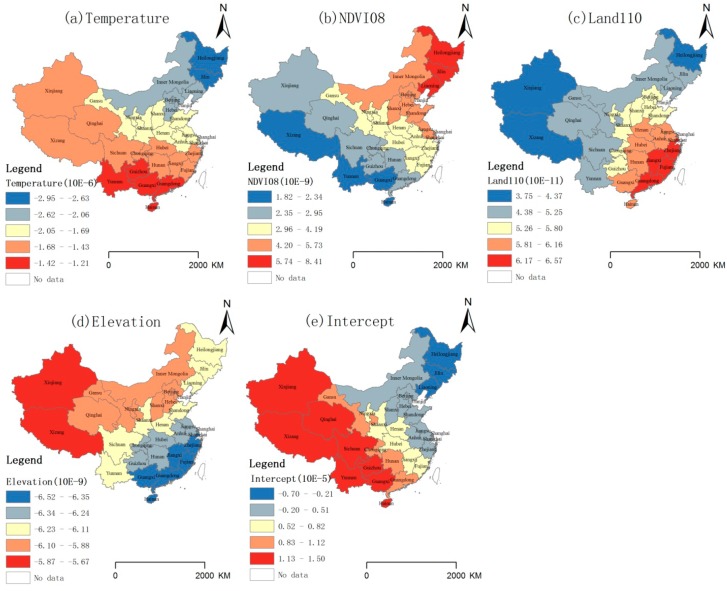
GWR model coefficients, 2006.

Taking the year 2006 as an example, the GWR model was analyzed (Figure 5 and Figure 6). According to the temperature distribution in 2006 (Figure 5a), the temperatures presented a declining trend from the south to the north, and except for Qinghai and Xizang, the temperatures were between 0–25 °C. Corresponding to GWR model temperature coefficient distribution (Figure 6a), temperature decreased the HFRS incidence (the coefficients were negative), and the absolute value of the coefficient presented a declining trend from the northeast to the southwest, which indicates the temperature constraints were more effective in the northeast compared with the southwest. The result indicates that, at a year scale, in 2006, the temperature rise led to HFRS incidence decrease, and this effect in Northeast China was more obvious than in the southwest. The result was consistent with study in Shandong [24].Temperature influences the rodent population by affecting the pregnancy rate, number of fetuses, birth rate, and survival rate. Higher temperatures restrict the number of rodents [36].

According to the NDVI08 distribution in 2006 (Figure 5b), Northeast China and Eastern China presented higher NDVI values than other areas. Corresponding to the GWR model NDVI08 coefficient distribution (Figure 6b), the NDVI of August for the previous year promoted the HFRS incidence (the coefficients were positive). Furthermore, the coefficient presented a declining trend from the northeast to the southwest, which indicates the promoting role of the NDVI of August for the previous year was more effective in the northeast compared with the southwest. The result was consistent with recent studies [38,39]. The NDVI of August for the previous year reflected the level of vegetation coverage, which was an indicator of food and living conditions for rodents in the winter epidemic time. The northeast China with higher NDVI08 value took a higher HFRS epidemic risk.

According to the Land110 distribution in 2006 (Figure 5c), this land use type is widely distributed in Northeast China, North China and West China, with a few regions in Southwest China. Corresponding to GWR model Land110 coefficient distribution (Figure 6c), Land110 promoted the HFRS incidence (the coefficients were positive), and the coefficient presented an increasing trend from the northwest to the southeast, which indicates the promoting role of Land110 was more effective in the southeast than in the northwest. According to the legend of land use, Land110 represents “Mosaic forest or shrub-land (50–70%)/grassland (20–50%)”, which may provide good habitats for rodents.

According to the elevation distribution in 2006 (Figure 5d), the southeastern terrain is lower than the northwestern part in China. Corresponding to GWR model Elevation coefficient distribution (Figure 6d), Elevation was protective against HFRS incidence (the coefficients were negative), and the absolute value of the coefficient presented an increasing trend from the northwest to the southeast, which indicates the elevation constraints were more effective in the southeast compared with the northwest.

Elevation presented a smaller constraint for HFRS incidence in the area below 1000 m than in other areas. And the result can be verified with Yan’s study [40], which proved that approximately 86.4% HFRS cases occurred in areas with 0–500 m elevation in the eastern part of China and the Sichuan Basin.

The intercept in Figure 6 epresented an increasing trend from the northeast to the southwest, which indicates the GWR model explains the HFRS incidence in the high-risk areas of Northeast China and North China better than in the low-risk areas of Northwest China and Southwest China. In the area with intercept below 0, there may be other factors reducing the incidence; and in the area with intercept above 0, there might be factors increasing the incidence, which should be explored in further study.

In our analysis, the optimal GWR model for 2005–2009 revealed that HFRS incidence was negatively correlated with temperature, precipitation, elevation, and positively correlated with humidity, NDVI01, NDVI08, Land110 and Land120. Temperature was negatively associated with HFRS incidence in 2005, 2006 and 2007. By affecting rodents’ rodent pregnancy rate, litter size, birth rate, and survival rate, temperature affects the HFRS incidence [5,38]. The appropriate temperature promotes rodent population growth, and high temperatures restrict the number of rodents [36]. According to Yu’s [41] and Tang’s [42] studies, 2007 was the warmest year on the historical record, which may have threatened rodents and therefore decreased the HFRS incidence. Precipitation was negatively associated with HFRS incidence in 2008 and 2009, and the result was consistent with the findings of previous studies [5,39]. Appropriate precipitation not only functions as a stimulus for plant growth, but also improves the bionergy and infection rate of the hantavirus, which eventually increases HFRS incidence. However, excessive rainfall could have a negative impact on rodents by destroying their habitats, and frequent rain may decrease the likelihood of rodent-to-rodent contact, rodent-to-human contact, and virus transmission due to decreased rodent activity and reduced human exposure [5]. Humidity was positively associated with HFRS incidence in 2009, which was consistent with Xiao’s studies [38,39]. The moist environment provided suitable conditions for rodents. NDVI was positively associated with HFRS incidence. NDVI reflects the level of vegetation coverage, which provides not only food but also shelters for rodents. The positive correlation of NDVI and HFRS incidence was proved in many studies [37,38].Yan’s study [10] showed that the highest correlation coefficient was 0.67 between three months backward from the NDVI and the number of cases of HFRS in farmland, which laid foundation for choosing monthly NDVI for analysis and also verified our results. Elevation was negatively associated with HFRS incidence in 2005, 2006 and 2007. According to Yan’s study, HFRS incidence significantly declined as elevation increased and the highest incidence was observed in areas with elevation of 100–200 m [6]. In Liu’s study [9], they found that DEM had a great impact on HFRS transmission in January, February, June and July, and that the risk of HFRS decreased with the increase of DEM. The negative effect of DEM for HFRS incidence is consistent with the mentioned studies. Land use is a traditional affecting factor for the rodents and HFRS incidence, which can be proved by many studies [38,40,43]. Land use provides different habitats for different rodents which adapt to various environments [43]. Since different studies were conducted on different spatial scales at different study area, the correlated land use types were different. Yan’s study [6] showed Timber forest and orchard land were appropriate environments for rodent hosts in China. In this study, HFRS was positively correlated with land use type: Land110 (Mosaic forest or shrub-land (50–70%)/grassland (20–50%)) and Land120 (Mosaic grassland (50–70%)/forest or shrub-land (20–50%)).

#### 3.4.2. 2010–2012: Spatiotemporal Heterogeneity Cause Analysis

The spatial auto-correlations of HFRS incidences in 2010–2012 were not significant; thus, the GWR model was not suitable for the analysis. Reasonable explanations for the HFRS incidence were explored. Because of the significant correlations between the HFRS incidence and Land50 and Land110, it made sense that Northeast China, which had more land use types of Land50 and Land110 distributions, had a relatively higher HFRS incidence.

Shaanxi simultaneously became the highest HFRS incidence area in 2010–2012. According to some studies [3,44], HFRS was mainly distributed in Huxian, Zhouzhi and Changan of Shaanxi [3]. According to Ma’s study in Xi’an, the dominant virus in Xi’an was HNTV and no SEOV was found, and there might be a ten-year cycle of HFRS in Xi’an, which can serve as a good explanation for the HFRS epidemic in Xi’an in recent years [45]. According to Li’s study conducted in Xi’an, the risk factors for HFRS included the workplace building site (near a rat’s nest), living at the edge of the village, and the presence of a river or pound around the workplace [3,44].The risk factors in this study explained the HFRS incidence in 2010–2012 to some extent. According to Barrios’s study [46], the increasing hantavirus incidence in recent years has been associated to global scale climate changes influencing the dynamics of forests and thereby inducing changes in rodents’ habitats, which might be the potential cause for HFRS epidemic in Shaanxi. Future studies should be conducted to clarify the potential cause.

## 4. Conclusions

In this study, the spatiotemporal heterogeneity of the HFRS incidence was analyzed, and GWR models were built based on the HFRS incidence data from 2005–2012 and the affecting factors. The findings suggested the chosen factors explained the spatiotemporal heterogeneity of HFRS incidence well for 2005–2009 and had better effects in Northeast China and North China than in the low incidence areas. At the same time, the chosen factors explained, in part, the HFRS incidence in 2010–2012. Regarding Shaanxi, which represents the highest HFRS risk province in recent years, environmental conditions, work conditions and mega-construction projects may have affected the incidence.

This study had some limitations. The spatial scale of the study was performed at the province level and the incidences were annual, which might miss or conceal the heterogeneity of HFRS incidences. Additionally, the absence of rat density and vaccination data, no distinguishing between the HNTV and SEOV, may have affected the explanations of the HFRS incidences. Future studies should make efforts to solve the mentioned limitations.

HFRS incidence demonstrated clear spatiotemporal heterogeneity in 2005–2012 and was primarily affected by meteorological elements (such as temperature and precipitation), landscape factors (such as NDVI and land use), and geographical factors (such as elevation). In recent years, the frequency of HFRS has been affected by human activities. Effective vaccination programs, rodent control measures and improvements in the living and work environments play important roles in HFRS control.
